# A Qualitative Study of Caregivers’ Experiences, Motivation and Challenges Using a Web-Based Mindfulness Intervention

**DOI:** 10.1007/s10597-019-00477-7

**Published:** 2019-09-30

**Authors:** Sigrid Stjernswärd, Lars Hansson

**Affiliations:** Department of Health Sciences, Lund University, Box 157, 221 00 Lund, Sweden

**Keywords:** Caregivers, e-Health, Mindfulness, Self-compassion, Web-based support

## Abstract

Caregivers report experiences of stress and burden that can affect their health negatively. Web-based mindfulness interventions have shown beneficial health effects in clinical and non-clinical populations, including caregivers. The study’s aim was to explore the experiences of a web-based mindfulness program, including motivation and challenges to use, in caregivers of a person with somatic illness. Ten participants were interviewed. Data was analyzed with content analysis, resulting in four categories illustrating the participants’ experiences of the program, including motivations and barriers to training: A timely or untimely intervention; Mainly positive effects even at low levels of training; Relationship to the patient; and Creating a routine and maintaining motivation. Qualitative studies can contribute to enrich our understanding of the value of such interventions, which may be a flexible supportive tool for caregivers. The findings illuminate the importance of supporting motivation and adherence to such interventions, with the potential for enhanced beneficial outcomes.

## Background

### Caregiver Burden

Around 42.1 million family caregivers provided care to an adult with limitations in daily activities at any given point in time in 2009 in the US (Feinberg et al. 2011). In Sweden, 1.3 million caregivers support someone regularly; out of those 900,000 are of working age and 140,000 have stopped or reduced their work time due to caregiving (Anhörigas Riksförbund 2016). Caregivers are at risk of negative stress (Adelman et al. 2014; Turcotte 2013), anxiety and depression (Rigby et al. 2009) due to caregiver burden and role overload (Adelman et al. 2014; National Alliance for Caregiving and AARP 2009; Turcotte 2013), whether supporting a significant other with a mental (Östman and Hansson 2004) or physical illness (Etters et al. 2008; Grunfeld et al. 2004; Rigby et al. 2009; Schrag et al. 2006; Schubart et al. 2008). Caregiver burden can encompass psychological, occupational and financial burdens (Grunfeld et al. 2004; Schubart et al. 2008). Caregivers express difficulties with finding own time, managing emotional and physical stress, and balancing work and family responsibilities (Longacre 2013). Supporting caregivers with e.g. stress management may help them deal more constructively with stress, with subsequent positive effects on psychological well-being (Guay et al. 2017). Lack of resources and energy, transportation issues and stigma can however be barriers to accessing help, which is why online interventions may be of value to increase accessibility to effective interventions.

### Mindfulness Interventions

Kabat-Zinn (2003) defines mindfulness as “the awareness that emerges through paying attention on purpose, in the present moment, and nonjudgmentally to the unfolding of experience moment by moment”. The literature shows evidence of mindfulness based interventions’ (MBIs) effectiveness in improving psychosocial and health outcomes for a variety of populations and contexts, including both non-clinical and clinical populations (de Vibe et al. 2012), both face-to-face (de Vibe et al. 2012; Keng et al. 2011) and online (Boettcher et al. 2013; Glück and Maercker 2011). There is a growing research interest in the efficacy of MBI’s effects for specific populations, including health care professionals and caregivers (Banerjee et al. 2017; Chiesa et al. 2017). Studies of patient populations and their caregivers (Birnie et al. 2010; Li et al. 2016; Longacre 2013; Stonnington et al. 2016) show beneficial effects of MBI (MBSR) regarding self-rated psychological symptoms (e.g. depression, anxiety, stress, mindfulness) (Li et al. 2016) and the potential to improve well-being among caregivers (Stonnington et al. 2016). The possibility to involve both patients and caregivers may also increase feasibility for caregivers that cannot leave the patient home alone (Li et al. 2016). In spite of the advantages of web-based interventions (e.g. availability, convenience of use) attrition levels can be high (Melville et al. 2010), which is why the exploration of factors supporting or deterring participants’ motivation and adherence to such interventions is necessary to enhance the potentially beneficial effects of such interventions.

Health professionals and caregivers are prone to experiences of chronic stress and burnout (Pinquart and Sörensen 2003; Spickard Jr et al. 2002; Sörensen et al. 2006), which can affect the body and brain negatively with effects on cognition, decision making, anxiety, mood and behavior (McEwen 2017). They may hence benefit from MBI’s effects on body–brain interaction and brain plasticity, with its subsequent positive changes in brain and body (McEwen 2017; Rasgon and McEwen 2016). Improvements in empathy and mental health outcomes also speak for such interventions’ value in enhancing emotional competencies in professionals dealing with others’ suffering (Lamothe et al. 2016). Self-compassion is more or less interwoven in MBI (Hölzel et al. 2011) and increased positive feelings towards the self through increased self-compassion can be noticed (Cairns and Murray 2015). This may be useful for caregivers that experience high levels of guilt, self-criticism, and interpersonal stress. Caregivers of a person with mental illness (MI) experienced the current web-based MBI as useful (Stjernswärd and Hansson 2016b). The research team customized the contents of the MBI program’s introductory and psychoeducational files to mirror the experiences of a wider group of caregivers, including caregivers of a person with somatic illness (SI). The current qualitative study is part of a larger project including a RCT that showed promising results (Stjernswärd and Hansson 2018).

The aim of the current study was thus to explore the experiences of the web-based mindfulness program, including motivation and challenges to use, in caregivers of a person with SI.

## Compliance with Ethical Standards

The project was approved by the Regional Ethical Committee, Lund, Sweden (dnr 2016/925). All individual participants included in the study filled in an online informed consent prior to their participation in the study. Information about the interview study was reiterated orally prior to the interviews. No remuneration was offered for participation in the study.

## Methods

### Intervention

The intervention consists of a web-based mindfulness program tailored for families living with MI or SI: its contents are related to caregivers’ situation and associated experiences of burden and stress. The program can be accessed through a computer/tablet/smartphone with Internet access. It contains audio/video files (960 min), including introductory information and training advice accompanied by written keywords on the screen, descriptive text files and instructions for daily mindfulness practice, a time log, and a private diary (not visible to the researchers). The recommended training time was set to 2 × 10 min/day, 6 days/week for 8 consecutive weeks. It includes basic mindfulness practices such as breathing exercises, body scans, mindful yoga/conscious movements, attention to experiences through the senses, and (self) compassion meditations (Table 1). The exercises remind of those in MBSR programs, except that they are kept to a maximum of 10 min/exercise to make them more easily practicable for participants with hectic schedules. The test period was set to 10 weeks to allow for individual flexibility. Weekly email reminders, including contact information to the researchers/technical support, were sent to the participants as reminders and motivators for training.

**Table 1:** The 8-week program’s 8 steps (English translation)

Week-wise contents (1 step per week)
(1) The breathing body
(2) Being present in the body
(3) Mindfulness in life and movement
(4) Compassion with the self - acceptance
(5) Wonderful pleasure
(6) Being whole
(7) Compassion with others
(8) To live with the possibility of choosing

### Participants

Participants in the project’s main study (RCT) (Stjernswärd and Hansson 2018) were recruited through advertisement in papers, newsletters, online, social media, and clinics/organizations with interests in caregivers. Information about the study and informed consent were available online. The inclusion criteria for participation in the project’s RCT were: age (> 18), being a relative/significant other to a person with MI or SI (mixed diagnoses/as reported by participants), having access to a computer/Internet, and to understand Swedish. The exclusion criteria were having prior experience of mindfulness meditation and an own severe MI that requires other professional treatment. In the current interview study, further purposive inclusion criteria were: having answered the RCT’s post-test assessments (T2) and having reported to be a relative of a person with SI. Of the 443 participants that fulfilled the inclusion criteria and registered to the main study using the online consent form, 398 answered the sociodemographic and baseline questionnaires (T1), after which all participants were randomized (http://randomizer.org/) into an experiment group (n = 196) and a wait-list control group (WLC, n = 202). Of the 136 and 73 participants in the experiment and the WLC group respectively that answered the T2 questionnaires, 13 and 11 in the respective groups who also reported being a relative of a person with SI replied “yes” or “maybe” to participating in a subsequent interview study when asked a T2. After the respective T2 assessments, the 24 participants were contacted by email and asked whether they still wanted to participate in the interview study. Out of these, 6 and 4 in the respective groups answered positively. One man and 9 women (9 partners, 1 mother) aged 25–73 (mean = 57.6 years), with training times ranging from 230 to 960 min (4 participants with training time 230–390 min, 1 with 530 min, 5 with 770–960 min), were interviewed. The training time was registered and retrieved online. All participants had a partner, all had a university/higher education but 1 (elementary school), 6 had an employment and 4 were not working, and all but 1 (sometimes) shared household with the patient. Time since diagnosis was 1–5 years ago (5 participants), 6–10 years ago (2 participants), 11–15 years ago (2 participants), and < 15 years ago (2 participants). Nine participants reported a good own physical health and one a very good one, while as many participants reported a good own mental health as a bad one (5/5).

### Data Collection and Analysis

As the intervention was web-based and participants were from multiple parts of the country, data was collected by the first author through phone interviews using a semi-structured interview guide developed for the current study. It included questions about the program’s contents and format, motivation and hinders to use the program, and general experiences of using the program, including potential effects of training and their consequences for the caregiver situation. Qualitative interviews were used to obtain rich descriptions of the participants’ experiences with the purpose of interpreting the meaning of the phenomenon of interest in the current study (Kvale and Brinkmann 2014). The interviews lasted 30–67 min (mean = 43). They were recorded and transcribed verbatim for analysis. Data was analyzed with content analysis (Graneheim and Lundman 2004). The transcripts were read several times to get an understanding of the whole material. Thereafter the first author underlined and coded meaning units answering the study’s aim by hand in the transcripts. The codes were condensed, compared and grouped into categories and sub-categories, based on similarities and differences between them. The transcripts were revisited and compared to the emerging coding scheme to make sure that the codes and categories covered all relevant data, without leaving out important data. The analysis included moving back and forth between the parts and the whole, and letting the informants’ voices speak, which is also illustrated by quotes in the “Results” section. The authors discussed the emerging results and reached consensus about the coding and findings.

## Results

### A Timely or Untimely Intervention

#### Caregiver and Life Situation

The participants described the intervention as a welcome initiative and their participation as more or less timely depending on their life circumstances and caregiver situation at the time of the intervention period. There was sometimes a paradox in terms of having a stressful situation and not managing to carry out the training when it was maybe needed the most. Illness fluctuations as well as general life circumstances could strongly affect stress levels and caregivers’ situation. Life events could contribute to more or less hectic times (e.g. move, work situation), either facilitating or hampering the training. Reflections were made about a reduced receptivity towards and possibility to pursue the training during tougher and more stressful periods, while at the same time potentially needing it the most. At a time of crisis connected to the patient’s deteriorating health status for instance, which also affected the participant’s own mental health negatively, the training engendered overwhelming feelings and had to be disrupted. On the other hand, caregivers experienced the training as valuable during stormy periods too, as it helped them cope more easily with the situation. When occurring during less stressful periods, adherence to the training was easier. Participants appreciated its effects. Simultaneously, they pondered over the fact that the calmer period also contributed to a more tranquil caregiver situation.

When one maybe needed it the most, it was most difficult to make the time. (P7)

In this situation, it was very good for me.//I could get away, lie there, and listen to his voice. (P10)

#### Expectations and Relevance

Participation in the study was an opportunity for caregivers to cater to their own needs, which motivated enrollment in the study. Expectations about the intervention, however, differed. While some participants were cognizant of relaxation exercises from previous experiences with such methods, and thus in some way familiar with what was awaiting them, others did not know much about mindfulness and were curious about it. They saw their participation in a research project as a motivating factor. Their participation in the study was viewed as both a valid excuse to make own time and as an occasion to take the training seriously; especially if focus had been on the needs of the significant other that was affected by illness more than on own needs for a prolonged time.

I appreciate these moments that were only for myself. (P1)

Belonging to the control group, however, appeared to be a potentially demotivating factor as this meant being on a wait-list with postponed access to the program. Some participants reported a certain hesitation or skepticism along with their interest for mindfulness. They valued the intervention’s positive effects and sometimes experienced them as unanticipated. Regardless of that, a few participants still had a perception of certain aspects of mindfulness as dopey. Others mentioned that mindfulness was now a fashionable trend in society and thus timely, which supported their training. Interestingly, all participants reported being willing or open to complete or get on with their training even after the intervention period.

The funniest thing is that I, in the beginning, was like… no, this is just one of these bluff things, it doesn’t work.//So it was extra fun to try it now.//And to feel that it works. (P3)

Several participants described the program as relevant for their circumstances, in the sense that it helped them address own needs and deal with issues specifically engendered by the caregiver situation, such as e.g. handling worry about the ill person and the caregiver role. Others described the program as useful on a more general level, e.g. to handle stress and worry, and to relax.

Once further into the training it was easier for me to let go of all the other things//that’s also what I was wishing for, not to dwell on things so much.//…that thing with my brooding and worrying for everything//It felt like it targeted these problems. (P7)

If one’s needs (e.g. for a therapist) and expectations on the intervention were not met, participants experienced the program’s contents as less relevant in relation to their caregiver situation, even if they could see potential general benefits of training. The experienced positive effects of training were significant motivators for training. So was its experienced potential as a coping tool to handle e.g. stress and difficult emotions. Most participants had recommended or could consider recommending the program to others in a similar situation, if one was willing to make time for it. Some participants mentioned that it might not suit everyone.

I had this relaxed feeling//and it motivated me to proceed with the training. (P6)

The participants’ understanding of the program’s structure at times affected training adherence. Not knowing exactly what was expected in terms of program structure and training dose (despite information), and lack of adherence towards the recommended dose sometimes induced a sense of insecurity as to whether one was doing the training properly and actually benefiting from it or taking it seriously enough. This could deplete motivation. The reduced ability to absorb information about dose and structure may have been due to stress, distraction or absent-mindedness, as reflected upon by the concerned participants. In contrast, other participants did not encounter such difficulties. They experienced the program as easy and clear to grasp both technically and content wise.

### Mainly Positive Effects Even at Low Levels of Training

#### Beneficial Effects of Training

All participants reported positive effects regardless of training time, even though participants with lower training time (as reported spontaneously during the interviews and in accordance with the time registered online) reported fewer/lesser effects than those with more training time—whether resuming the training for health reasons, lack of time or motivation, unmet expectations or other unreported reasons. Participants saw the training as an occasion to look after own needs and as a valid excuse towards the caretaker to make own time. They experienced this opportunity and the training itself as relaxing, calming and sleep promoting. Several participants reported falling asleep during training. This made some participants question the accuracy of their training habits and its potential value. Nevertheless, they generally interpreted this as a need to relax.

So I told him, now you have to be quiet because I am concentrating, I am doing my exercises//it was a way for him too to accept that I was lying down.//I think I feel guilty//…I create strange rules//it made it easier to change that. (P3)

After a few weeks, I felt//ah, that’s agreeable…//to get away for a while and do it. (P7)

The training made it easier to let go of tendencies to worry. It helped manage one’s thoughts, feelings and habits by spending less energy on things that one now could see were beyond one’s control to change. The training helped focus and enjoy the present moment, rather than ruminate about the past or worry about the future. Participants reported feeling calmer, more anchored and harmonious. They experienced an expanded inner space and stability. This facilitated handling stress and difficulties in daily life, and encounters with others and others’ needs.

If the me is in the stomach somehow, it’s small and often vulnerable. But when I do one of these three things (work out, yoga or mindfulness), it gets bigger and more stable. (P4)

If one gets more attention and reaches a calmness, and can handle the situation differently, it gets better. (P8)

The training helped illuminate automatic patterns of thoughts, feelings and behavior. An enhanced awareness of reactions to triggers contributed to an improved ability to act rather than react, e.g. by becoming less alarmed by potential stressors. It paved the way for making a halt, reflecting upon the situation and breaking automatic reactions.

I feel calmer//It feels as if I reflect more before I get going, so to say.//I think more before I act… do I really need to worry about this? (P3)

It facilitated the consideration of alternative approaches to potential problems. It supported a change of perspective, opening up the participants’ senses and mind to other impressions and people. This in contrast to an earlier tunnel vision with focus on difficulties and daily stresses. Participants reported an increased awareness of positive aspects in life. This was especially valuable during tougher periods.

//one accepts that it’s the way it is now, and that we now need to find something more constructive than getting stuck in the negative.//Both my husband and I experienced that it made things simpler. (P1)

Tendencies to (self) judge became visible. The training facilitated a more accepting and (self) compassionate attitude and approach towards own and others’ feelings and prerogatives. The training became an opportunity and help to better balance own and others’ needs. It led to an increased self-knowledge and awareness about ways of relating to the self and others. Through the training, participants detected tendencies to adapt to others’ needs (true or imagined) and to react automatically and emotionally on triggers.

When one does things day after day//or things one is expected to do, or thinks one should do, one has one kind of life. But when one makes a halt and does a thing like this (mindfulness), one discovers that…//there is a possibility for another existence. (P4)

I am more content with life, well, with myself and it changes things… because otherwise, one always tries to fulfill others’ needs. Now I try to fulfill my own instead. (P3)

#### Negative Effects of Training

The most serious negative effect of training was reported by a participant in crisis, which led to anxiety and stressful feelings that were reinforced by the training. “Keeping the lid on” was a temporary coping strategy to handle the caregiver situation, which the training did not allow. This is why the participant chose to end the training at that time. Nevertheless, the same participant reported positive effects and (s)he wished to pursue the training once the crisis had abated. The most frequently reported negative effect was experiencing the training as another stressful demand in daily life. Inability to train according to the recommended dose induced a sense of stress. Participants worried about not being able to complete the program within the planned intervention period. Some participants experienced this as a personal failure. It also raised concerns about “letting the project down”.

I am never going to make the training in ten weeks, how will it go? (P4)

### Relationship to the Patient

Whether the training had any effect on the caregivers’ relationship to the patient was sometimes difficult to determine for the participants. It could be difficult to separate cause and effect, e.g. due to health fluctuations in the patient and/or an improved life and/or caregiver situation. Nevertheless, several participants felt that the positive effects of training had subsequent positive effects on their caregiver situation and relationship to the patient. An enhanced inner stability and harmony made it easier to handle stress, not to react on autopilot or react emotionally to stressors and negative feelings, and to meet and address others’ needs in a less judgmental, more compassionate and constructive way. It helped separate the condition from the patient, to see the person behind the symptoms, and contributed to being more understanding and patient. It aided in becoming more honest and clear in one’s communication, addressing both own and others’ needs.

Yes, naturally (effect on relationship), in the sense that I don’t get angry in the same way. (P6)

Then I was calmer and a bit more compassionate. (P7)

Meanwhile, other participants could not discern any specific effects of training on their relationship to the patient, but rather potential general positive effects for them as a person. Participants with a shorter training time also referred to the shorter training time as a potential reason for lesser effects.

Perhaps not my situation as a caregiver, but it has affected me as a person maybe. (P8)

Some participants were open with their training, sharing their experiences with the patient and family members and occasionally doing some of the exercises together with the caretaker. Others kept it private, which felt essential and possible thanks to the web-based format.

### Creating a Training Routine and Maintaining Motivation

#### Creating a Routine, More or Less Challenging

Creating a daily training routine came through as easy for some participants and difficult for others. This partly depended on one’s general life situation, with potentially stressful life events, and other competing commitments. Participants mentioned facilitating or hindering factors. Examples were availability of time in relation to work (e.g. whether working vs. being on holidays or retired), whether one could do the training undisturbed (at home, at work, or when commuting or traveling), whether it was made a priority or put off, and whether it was remembered. Potential barriers were technical difficulties with the program (e.g. interruptions due to a failing Internet connection), insecurity about the training dose and unmet expectations, which reduced motivation and adherence to the training.

It takes time to create a routine. (P8)

In the beginning, I had to remind myself. But now, now it has become more like a routine. (P1)

Some participants experienced the training time as adequate and practicable, while others described it as challenging to fit into daily life. Being successful with establishing daily training routines facilitated adherence to the recommended dose, while still allowing flexibility to skip or redo a meditation exercise depending on the circumstances and prerequisites to do the training.

For me it’s good. That they’re short (the meditations)//because it’s easier to integrate into daily life. (P10)

It was too difficult to uphold the pace. (P5)

#### A Flexible and Accessible Intervention

The program’s flexibility and ease of use facilitated its use. The program was accessible wherever one had a mobile phone/tablet/computer with an Internet connection. This enabled the training whenever and wherever it felt convenient. Most participants felt at ease with the web-based format, which was a prerequisite to participate and do the training. It did not require going somewhere special at a certain time. The latter was not considered as a viable alternative; the participants said that it would be too energy and time consuming and, for some, difficult to leave the patient.

It’s feasible wherever and whenever one wants. (P2)

The majority reported not missing the support from a live instructor. The recorded voice was a sufficient guide. Many participants appreciated the instructor’s voice while a few did not. Some participants wished for the possibility to receive feedback on their training (e.g. on whether it was done properly, or on how to keep up with the training) and on its effects. This to better understand the rationale behind mindfulness, to work as a motivator, and to hinder resuming the training, for instance when puzzled about the effects of training (e.g. falling asleep, emerging sadness).

It might have been useful, at a few occasions//to talk to a real person, to regain motivation,//in a more tangible way know how to handle it and make time for the training//. (P5)

The weekly emails reminded participants about the availability of the research team (via phone/email) for enquiries and technical support. The project website’s Q&A section (to which a link was included in the weekly emails) also addressed potentially occurring issues. Some participants reported getting clear answers and adequate information from the project website/emails. A delay in the program’s training time registration function prevented some participants from moving forward in the program if they switched it off before the training time was registered. This caused frustration and a sense of insecurity about the program’s further contents, at times depleting motivation. This technical issue was addressed in the project website’s Q&A section, as some participants mentioned, but the information may have gone unnoticed by some participants.

#### Repetition and Familiarity as Motivators and Hinders

Many participants commented upon the program’s repetitive aspects, both in regards to the instructions and the exercises. Some participants mentioned that hearing the instructions (how one should sit/lie down/stand) in the beginning of every meditation could be dull or distracting (for instance when several options were possible, e.g. to sit or lie down). When it came to the repetitive nature of the exercises, the participants sometimes initially experienced it as monotonous. Eventually, they viewed it as positive as the by then well-known exercises felt familiar, safe and settled, but also as new things were discovered along the training while repeating the same exercises. Participants experienced that the effects became gradually more immediate.

It oughtn’t be new or varied, one should do it again and again, because it settles//when it’s the same meditation, one discovers oh… I hadn’t heard that//…one discovers new things after a while. (P1)

One could see a risk for monotony if pursuing the training with the same program after the intervention period. Participants thus suggested a variation in wordings to maintain interest in and curiosity about the exercises.

#### Suggestions to Boost the Program

While several participants expressed undivided satisfaction with the program (contents/technique) and experienced it as easy to use, some participants made suggestions to increase its attractiveness, relevance and usability. Examples were more silent periods during the exercises, allowing time to settle and meditate in silence; fewer repetitions of the initial instructions with every exercise; and the possibility to pause and back in the audio files (e.g. if the meditation was disrupted for some reason, or one wanted to re-listen to parts of the same track). Further suggestions included a variation in wordings, and more about the mindfulness rationale, which participants experienced as motivating for training. Participants appreciated the weekly advices (audio files) for this very reason.

He (the instructor/voice) could pause. So one winds down.//I would recommend a silent moment. (P10)

The advice, yes, I thought it was rather motivating.//One got a better understanding… yes, this was what this exercise was for, what one should think about… (P7)

Some participants suggested the inclusion of additional program contents, further illuminating their needs as caregivers. Examples were even more psychoeducative information about common feelings and experiences in caregivers, information about “co-dependency” as described by the participants, or contact with a therapist. Nevertheless, they realized that such contents might have been beyond the scope of the current intervention. Most participants experienced the weekly emails as neutral or as positive reminders for training. Participants discussed the availability of an additional short tutorial or email reminder, to work as an aide-mémoire and easy overview of the program set-up and training dose in cases of insecurity.

## Discussion

Keeping in mind the limited sample size, the reported effects are nevertheless similar to those described in earlier studies, which points to the potential value of web-based MBI to help caregivers cope with stressful life circumstances. Effects includes an enhanced awareness (Cairns and Murray 2015; Long et al. 2016), self-acceptance (Cairns and Murray 2015; Colgan et al. 2017; Finucane and Mercer 2006; Mitchell and Heads 2015; Wyatt et al. 2014) and sense of competence, control, coping and/or choice (Cairns and Murray 2015; Colgan et al. 2017; Long et al. 2016; Mitchell and Heads 2015; Moore and Martin 2015; Wyatt et al. 2014). Not only can MBI affect how one relates to the self and one’s own thoughts and feelings, but also, as seen in previous studies (Bihari and Mullan 2014; Birnie et al. 2010; Long et al. 2016; Mitchell and Heads 2015) can it affect how one relates to others. This can help improve communication and interactions with others (Bihari and Mullan 2014; Long et al. 2016), contributing to being more present in relationships and asserting both own and others’ needs (Bihari and Mullan 2014); a central issue, as also seen for participants in the current study. Some participants, however, did not experience any effects on their relationship to the patient. The benefits of qualitative studies for exploring interpersonal aspects of mindfulness have been pointed out (Sauer et al. 2013) and also their potential to illuminate processes that can’t be captured through quantitative studies (Colgan et al. 2017), further motivating the use of varied methods to investigate experiences with MBI.

Adequately addressing caregiver needs may contribute to enhance adherence to supportive interventions and reduce attrition rates. Caregiver needs may also fluctuate depending on the patient’s health status and other life circumstances, which ought to be taken into consideration when developing supportive interventions. MBI can contribute to an enhanced ability to deal with others’ suffering through increased empathy, emotional acceptance and identification of both own and others’ needs, potentially preventing mental health deterioration in persons dealing with others’ suffering (Lamothe et al. 2016), as also seen in the present study. Through processes such as the identification, acceptance and balancing of both own and others’ needs and suffering, MBI can help caregivers cope with their situation. As also seen in previous studies (Hölzel et al. 2011; Wyatt et al. 2014) processes such as an enhanced awareness of triggers and automatic reactions, and an increased attentional and emotional regulation, can help respond rather than react to stressful situations. MBI can help deal with negative emotions and prevent automatic tendencies to jump to negative conclusions (Finucane and Mercer 2006). This may help avert rumination and negative spirals of thoughts and feelings, which seem to be common issues among caregivers.

Caregivers are at risk of own health problems and report needs of support, which motivated the participants to enroll in the current study. MBI have beneficial effects for healthy and clinical populations, including persons with anxiety and depression (Boggs et al. 2014; Finucane and Mercer 2006; Langdon et al. 2011). Nevertheless, such interventions can be counter-indicated, for instance in connection with serious mental health problems. A crisis afflicting a participant in the current study led to the decision to terminate the training. The emergence of troubling thoughts and feelings and the exacerbation of anxiety and depression have been reported previously (Lomas et al. 2015). Wyatt et al. (2014) pinpoint the importance of informing participants about what to expect or not from interventions, but also about the potential for distressing experiences. This was also done in the current project. In case of overwhelming disquieting thoughts and feelings, participants were recommended to pause the training and/or seek professional support. The research team was available for contact. While adverse effects are not commonly reported in the literature (Li et al. 2016), the current study also shows that difficulties with adhering to the recommended training does can induce feelings of stress and personal failure. Life events and stress levels affected the participants’ receptiveness towards and ability to adhere to the recommended training dose. While potentially needing it the most, it was sometimes difficult to make the time and uphold training motivation. Enhanced clarity about the potential of web-based interventions to meet specific caregiver needs may help address the issue of expectations. Realistic expectations may further prevent participant disappointment, motivation drops and dropout.

Motivation and adherence in web-based interventions are known challenges. Facilitating factors included the program’s flexibility and ease of use, beneficial effects of training, participation in a research project, and the wish to address one’s situation as a caregiver. The match between MBI and the participants’ needs and expectations can affect motivation, similarly as understanding the program’s rationale, which goes in line with previous studies on web-based interventions (Banerjee et al. 2017). As also seen in previous studies (Boggs et al. 2014; Cairns and Murray 2015; Moore and Martin 2015; Stjernswärd and Hansson 2016b) conflicting time pressures and inflated expectations can deplete motivation and impede on training. So can the occurrence of technical glitches, and insecurity related to the intervention’s contents and format, confirming previous research (Schneider et al. 2014). A positive attitude, being open-minded, realistic and curious while accepting the limitations of MBI can help maintain motivation (Banerjee et al. 2017; Cairns and Murray 2015). This further illuminates the importance of clear information and managing expectations related to supportive interventions. Therapy guidance may help improve adherence and thus treatment outcomes, although it may be costly and restrict scalability. This is why Spijkerman et al. (2016) suggest additional automated support, e.g. through automated text messages and personalized experience stories that can illustrate dealing successfully with the challenges of training. The group’s normalizing and validating value has been mentioned in group-based MBI (Cairns and Murray 2015; Finucane and Mercer 2006; Wyatt et al. 2014), which the current intervention lacks. The possibility to customize interventions according to individual preferences, e.g. through the optional inclusion of live (face-to-face or in virtual rooms) support and exchange of experiences, or automated feedback, may potentially enhance satisfaction with the program and hence benefit more users.

### Strengths and Limitations

Even though the interviews generated rich data, the sample was limited. When recruiting participants, it was stated that the participants’ experiences were valuable regardless of training time, whether one had completed the whole program or not; this as an attempt to reach out to participants’ with varied experiences of the program and to avoid bias towards only positive experiences. The participants’ training time was varied, possibly contributing to a richer understanding of reasons to keep up with or end the training. There was a majority of women and highly educated participants, also limiting transferability of the results. This seems common in web-based interventions of this nature (Stjernswärd and Hansson 2016a; Whitebird et al. 2011). The recruitment of the total project sample may also have been biased towards individuals that are more prone to seeking help actively.

## Conclusions

Qualitative studies can contribute to enrich our understanding of the value of MBI for caregivers. Both beneficial and negative effects of training were illuminated. The findings show that caregivers can benefit from such interventions and areas for enhancements were identified. The identification of motivators and barriers to training can contribute to a better understanding of the participants’ reasons to pursue or discontinue their training, and to better customize such interventions. This may contribute to enhancing adherence and motivation to MBI and thus their potential beneficial outcomes.
